# Respiratory findings are not less frequent in food allergic children

**DOI:** 10.1186/2045-7022-1-S1-P98

**Published:** 2011-08-12

**Authors:** Derya Altintas, Gulbin Karakoc, Seval Kendirli, Mustafa Yilmaz, Dilek Dogruel

**Affiliations:** 1 Cukurova University,Faculty of Medicine, Pediatric Allergy and Immunology, Adana, Turkey

## Background

Food allergy (FA) has increased dramatically in recent years and it is now recognized as worldwide problem and accounts for a bread spectrum of disease. Although food induced respiratory symptoms are less frequent, their presence, usually are associated with other symptoms. In this report we evaluated respiratory and other clinical outcomes during food challenge in children.

## Patients and method

136 children with the positive food challenge that were on follow up in Cukurova University, Pediatric Allergy and Immunology Division were enrolled to the study. Clinical history, physical examination, skin prick test and food specific IgE levels and clinical outcomes during food challenge were evaluated in all children.. Open food challenge was performed in children younger than 2 years or in older children who refused the food because of different tastes.

## Results

There were 56 girls (41.1%) and 80 boy (48.9%) with the mean age of 38.9±23.9 months. Cow's milk was the most common allergen in all ages (57.3%) and followed by egg white (30.8%), wheat (17.64%) and peanut (5.1%). Skin reactions (urticaria,eczema) were the major symptoms occurred during food challenge and identified in 96 cases (56.5%). As the second most common symptoms, upper and/or lower respiratory tract symptoms were observed in 62 patients (53.4%) and 12 patients (9.2%) showed gastro intestinal symptoms . Of the positive allergen provocation, 78(57.35%) were immediate type reaction and 20(14.7%) late-onset reaction. 38 patients (27.95%) had combined reactions. 22 patients with respiratory symptoms developed inhalant allergen hypersensitivity (35.48%).

## Conclusion

In this study we found food induced respiratory symptoms more frequently compared to the previous studies. These patients should be followed up for the development inhalant allergen hypersensitivity.

